# Comparative study and dyeing performance of as-synthesized azo heterocyclic monomeric, polymeric, and commercial disperse dyes

**DOI:** 10.55730/1300-0527.3484

**Published:** 2022-07-03

**Authors:** Shehu Sa’ad ABDULLAHI, Haruna MUSA, Shehu HABIBU, Abdullahi Haruna BIRNIWA, Rania Edrees Adam MOHAMMAD

**Affiliations:** 1 Department of Polymer Technology, Hussaini Adamu Federal Polytechnic, Kazaure, Jigawa, Nigeria; 2 Department of Pure and Industrial Chemistry, Bayero University, Kano, Nigeria; 3 School of Chemical Sciences, Universiti Sains Malaysia, Penang, Malaysia; 4 Department of Chemistry, Federal University Dutse, Jigawa, Nigeria; 5 Department of Chemistry, Sule Lamido University, Kafin Hausa, Jigawa, Nigeria; 6 Department of Sciences, Faculty of Education, Open University of Sudan, Khartoum, Sudan

**Keywords:** Coupling components, carrier dyeing, terasil brilliant violet, terasil scarlet, brown, monomeric, polycondensation

## Abstract

In this study poly (4-nitrophenylazo-3-aminopyridine - formaldehyde) (PNAAP-F) and poly (4-nitroarylazo-3-chloro-6-hydroxypyridine - formaldehyde) (NAACHP-F) were synthesized via diazotization, coupling and polycondensation reactions. The structural properties of the as-synthesized dyes were acquired using Fourier-transform infrared spectroscopy (FTIR) and UV-visible absorption maxima and their color, yield, melting point, solubility, and viscosity were determined via standard methods. UV-visible and FTIR results show successful formation of the polymeric dyes due to shift of wavelength of maximum absorption (*λ*_max_) (440–490 nm, 480–540 nm) and new absorption peak at around (2780–2995 cm^−1^) for methylene bridge respectively. The dyes were found to be of good yield (monomeric: 73.3%–87.2 %, polymeric: 53.8%–76.6 %), low melting point (monomeric: 112.6–121.2, and 136.0–137.0 °C, while polymeric: 134.0–144.5, and 149.4–154.7 °C), soluble in some solvents. The dyeing activity was carried out and assessed on nylon and polyester fabrics using the standard methods. The dyeing process was carried out via high temperature and carrier dyeing methods. The dyeing properties of the synthesized dyes were compared with those of commercial disperse dyes (terasil brilliant violet and terasil scarlet, brown). The dyeings of nylon and polyester had a very attractive hue and the color ranges from yellow and deep yellow shades with very good to excellent fastness to light, washing, hot pressing, and rubbing.

## 1. Introduction

Polymeric dyes are a novel form of dyes in which organic dyes are chemically incorporated into the main or side chains of polymer [1]. Polymeric colorants have acquired popularity, applicability, and acceptance as a viable alternative to traditional colorants (dyes and pigments) [2]. The marriage of polymer and dye chemistry enables the chemist to design unique materials that exploit the best characteristics of both pigments and dyes [3]. The synthetic dyes play the major role in textile fiber fashioning [4], specialty materials having several advantages over their monomeric dyes counterparts, consequently in the last few decades; they have been documented well by many research scientists [4]. They have been widely applied on fiber because of certain essential dye properties such as fast leveling, fastness to light and wet treatment, low sublimation rate, and very good thermal and chemical resistance [5]. Habibu et al. studied the synthesis, characterization, and application of some polymeric dyes derived from monoazo disperse dyes [6]. The dyes generally have a good exhaustion in both fabrics, while nylon exhausts better than polyester. Their fastness ratings on nylon and polyester were satisfactory, with the polymeric dyes having more brilliant colors in general. Polymerization of prospective monomeric dyes on nylon and polyester results in increased shade brilliancy as well as improved fastness capabilities [6]. Polymeric dyes have excellent brightness and extremely fadeless [7]. Colorants, which include chromophores of dyes usually consisting of C=C, N=N, C=N, attached to aromatic or heterocyclic rings, containing oxygen, nitrogen or sulfur, have been widely used as dyes owing to their versatility in various fields and high technology, including textiles, paper, leather, plastics, biological staining, lasers, liquid crystalline displays, ink-jet printers, and in specialized applications, such as food, drug, cosmetic and photochemical productions [4,5]. Dyes are classified based on their chromophoric groups or method of application unto the substrate. Azo dyes are the most studied group of synthetic dyes [8,9]. Li et al. reported the coloration of calcium alginate fiber with dye and auxiliary derived from polyvinyl amine (PVAm). I. complex dyeing with polymeric dyes containing PVAm. The result of dyeing and fastness rating reveals that the better washing and rubbing fastness were achieved because of the strong chelating structure between fibers and dyes molecules [10]. Abdullahi et al. reported the facile synthesis and dyeing performance of some disperse monomeric and polymeric dyes on nylon and polyester fabrics, their finding showed that polymeric dyes have higher washing and light fastness (grade 8) than monomeric (grades 4 and 6) and commercially available dispersion dyes (grades 4 and 6). This is due to increased conjugation, which leads to the formation of large molecular weight compounds [11]. Shan et al. studied synthesis and dyeing properties of polyvinyl amine dyes for cotton. In their study the fastness rating was assessed, and the results indicates good wash and dry rub fastness grades of 4 and 4–5, respectively, and also 99% dye-fiber fixation [12].

Azo dyes are widely used in the textile industry and are the largest and most versatile group of synthetic organic dyes, with a tremendous number of industrial applications [13]. Azo dyes are characterized by the presence of one or more azo group (-N=N-) in association with two or more aromatic or heterocyclic system, in which many heterocyclic compounds are used extensively in disperse dye preparation for textile or nontextile applications [5,14]. These dyes are now marketed to produce a full range of dispersed dyestuffs without the use of colorants based on heteroaromatic diazo components [15]. The interesting future of azo dyes-based materials is the presence of cis-trans azo bond photoisomerization. Switching between the *trans* and *cis* isomers is possible by irradiating the molecules with light at specific wavelengths. It is also known that the position and the shape of the characteristic absorption bands of π–π* (more intense for the trans isomer) and n–π* (more intense for the cis isomer) character depend strongly on the nature of the substituents [16]. The color intensity increases as a result of trans–cis photoisomerization [17]. Most of the heterocyclic dyes are derived from the diazo and coupling components consisting of five-membered rings containing one or more nitrogen or any heteroatoms, with the rings being fused into another aromatic ring or in some cases six membered ring containing one or more nitrogen or any heteroatom [7,18]. Heterocyclic coupling components are aromatic compounds that contain heteroatom in their molecules (S, N, O) [19]. The azo dyes containing heterocyclic rings result in brighter and often deeper shades than their benzene analogs [20]. On the other hand, they are very important in applications such as disperse dyes for polyester fibers, reprography, functional dye and nonlinear optical systems, photodynamic therapy, and lasers [14, 21].

The as-synthesized monomeric and polymeric dyes have a moderate planar molecular structure that can easily penetrate and remain fixed in the matrix of polymeric nylon and polyester fabrics [9]. The dyeing affinity, fixation, substantivity and fastness properties increase on the nylon and polyester fabrics dyed with polymeric dyes in comparison to the monomeric, and commercial disperse dyes due to the increase in the conjugation between dye molecules and intermolecular hydrogen bonding between the dye molecules polar groups within the fiber and also between dye molecules and fiber [8,9]. This research work aims to harness the potential utilization of formaldehyde, aqueous oxalic acid, and heterocyclic coupling components together with azo chromophore to produce a disperse polymeric dye with excellent dye-fiber interaction and fixation. The designed heterocyclic azo polymeric dyes D_1_ and E_1_ are moderates and planar molecules that can be easily absorbed into the space of the fiber polymeric chains and remain fixed, due to the formation of high molecular weight dyes and hydrogen bonding that exist between dye molecules and polar group of the nylon and polyester fibers which leads to excellent dye-fiber fixation and fastness properties. The use of formaldehyde and aqueous oxalic acid during polymerization is to form a bridge (used as crosslinkers) between two or more monomeric dyes. In this study, new class of heterocyclic azo disperse polymeric dyes was prepared from polycondensation of their corresponding monomeric counterparts which were obtained through diazotization and coupling reactions. The as-synthesized heterocyclic azo monomeric dyes were subjected to polycondensation reaction in the presence of formaldehyde and aqueous oxalic acid to yield heterocyclic azo disperse polymeric dyes. The properties of as-synthesized dyes were acquired via FT-IR, UV-visible, percentage yield, melting point, solubility, and viscosity techniques. The as-synthesized dyes and two commercial disperse dyes (terasil brilliant violet and terasil scarlet, brown) were applied on nylon and polyester fabrics using carrier and high-temperature dyeing techniques and their substantivity on these fibers was assessed using standard methods.

## 2. Material and methods

### 2.1 Materials

4-nitroaniline (Sigma-Aldrich, St. Louis, MO, USA), 2-aminopyridine (Sigma-Aldrich), and 5-chloro-2-pyridinol (Sigma-Aldrich), sodium nitrite (Sigma-Aldrich) and HCl (Sigma-Aldrich) were used in the synthesis of the monomeric disperse dyes. Aqueous oxalic acid (Sigma-Aldrich) and formaldehyde solution (37% w/v) (Sigma-Aldrich) were used as crosslinking agents to produce the polymeric disperse dyes, polyester, nylon fabrics (Nigerian Institute of Leather and Science Technology Zaria).

### 2.2 Synthesis of diazonium salt by diazotization

The diazo components were synthesized using the standard procedure as reported by Habibu et al. [6]. The 4-nitroaniline (7.65 g, 0.06 mol) was dispersed in distilled water (20 mL) contained in a beaker that stands in an ice bath. As much as 10 mL of concentrated HCl was added to this solution over a period of 2 min while the dispersion was stirred with a magnetic stirrer. The temperature of the ice bath was maintained at 0–5 °C by the addition of common salt (NaCl). Sodium nitrite (4.3 g, 0.06 mol) was dispersed in distilled water (20 mL) and added dropwise to the stirred suspension over a period of one hour to attain complete diazotization.

### 2.3 Coupling reaction

Coupling component (0.06 mol) was suspended in distilled water (20 mL) contained in a 250-mL beaker at 0–5 °C in an ice bath. The diazonium salt solution was added dropwise to the coupling component over a period of 10 min while continuously stirring. The stirring was continued for further 45 min as the dye crystals precipitated. The dye was filtered, washed with water, and recrystallized in acetone [22]. The dyes were weighed, and their percentage yields were calculated using the formula [23].


(1) $$\%\;\text{yield}=\frac{\text{weight of the dye}}{\text{theoritical yield}}\times 100\%$$

### 2.4 Polycondensation of monomeric dyes by crosslinking reaction

Monomeric dye (0.05 mol) was suspended in distilled water (50 mL) and then 37% w/v formaldehyde solution (0.5 mL) and oxalic acid (0.5 g) were added. The reaction mixture was gradually heated to 90–95 °C and maintained at that temperature for 1 h. The reaction mixture was cooled, filtered, and washed with water until the filtrate was neutral. Finally, it was washed with 20 mL methanol and air-dried [23].

### 2.5 Characterization

The solubility of the dyes was carried out according to the method adopted from [12]. The solubility of each sample was carried out in a variety of solvents which include water, ethanol, methanol, chloroform, CCl_4_, dichloromethane, petroleum ether, and acetone. As much as 5 mL of each solvent was added to about 0.1 g of the sample in a test tube and shaken vigorously. The solubility was carefully observed and recorded. The melting point of each dye sample was determined using a microprocessor melting point apparatus (WRS-IB) model (England). The samples were grounded into a fine powder and the one-end-closed melting point capillary tube was filled to half with the powdered dye sample and placed in the machine. The samples were heated, and the melting range was carefully observed and recorded. The viscosity of synthesized dyes was acquired using Ostwald capillary viscometer (England). The solution containing 1.0 g, 0.75 g, 0.50 g, and 0.25 g of each dye sample in 100 mL of acetone was prepared and used in the measurement [24]. UV-visible spectroscopy was used to obtain the wavelength of maximum absorption of the as-synthesized dye samples using New Line medical instrument (England) (Spectrum Lab752S) UV-visible spectrophotometer in the region between 400 and 600 nm. The Agilent Technology (Cary 630FTIR) Fourier-transform infrared (FTIR) spectrophotometer (USA) was used [25]. The spectra of all the synthesized monomeric and polymeric dyes were acquired within the frequency range of 4000–600 cm^−1^.

### 2.6 Dyeing process

#### 2.6.1 High-temperature dyeing technique of nylon and polyester fabrics

High-temperature dyeing was carried out using a domestic pressure cooker (China). As much as 0.25 g of each dye together with the same quantity of detergent was taken in 50 mL of distilled water. Then, 2 g of each fiber was placed in the pressure cooker and the temperature was raised to 120 °C and maintained for about 1 h with slow and continuous agitation. The fiber was then removed, rinsed with detergent, water, and dried [26].

#### 2.6.2 Dyeing of polyester and nylon using carrier dyeing technique

As much as 0.1 g of finely divided dye sample was dissolved in 10 mL ethanol. About 30 mL of distilled water was added and the dye solution boiled to remove the ethanol. The solution was filtered, and 2 mL of dispersing agent (Dispergator SM-SN) solution was added followed by the addition of 6 mL of a carrier, 1-methylnaphthalene, and the solution made up to 100 mL by addition of distilled water. Then, 2 g of the fiber (nylon or polyester) was placed in the dye bath at 60 ºC. The temperature was raised within 90 min and the dyeing process continued at the mentioned temperature for further 30 min. The dye bath was cooled, and the dyed fiber was thoroughly washed with hot and then cold water. Finally, the unfixed dye molecules and the dye assistants was removed by treating the fiber for 30 min at 70 ºC in a solution containing sodium hydroxide (2 g/L), sodium dithionite (2 g/L), and 0.1 % dispergator [23].

#### 2.6.3 Light fastness

The light fastness properties of the dyed samples were assessed using the standard procedures ISO 105-BO2:1988 method. It was done on a nonnatural light fastness tester MK1 tailored with mercury-tungsten (MBTF) 500-watt lamp. The samples were exposed simultaneously with blue wool values for 96 h and assessed [26].

#### 2.6.4 Rubbing fastness

The dyed fabric piece was scrubbed on a smooth dry white cloth and later observed. The staining of the adjacent white cloth was observed and assessed in agreement with the test method provided by ISO 105-X12: 2001 method [27] (dry and wet) standard.

#### 2.6.5 Hot pressing fastness

Dry, damp, and wet pressing fastness tests were carried out according to the standard method ISO 105-POI 1993 method [27]. The dyed fabric (5 × 4 cm) was sandwiched between two pieces of dried white cloth to form a composite specimen. Hot pressing iron was then placed on top and allowed to stay for 15 s. The degree of staining of adjacent white cloth was then assessed using greyscale.

#### 2.6.6 Wash fastness

The test for color fastness to washing was determined using the ISO 105-CO6:2010 test method [28]. The fastness of the dyed washed samples was assessed using the standard methods. The samples were cut to 5 × 4 cm and placed in between two pieces of undyed materials with the same size. These samples were sewed together to produce a composite sample. The composite samples were distinctly absorbed into washing liquor comprising 100 mL of 4 g dm^−3^ detergent solution and disturbed for 30 min at 50 °C. The samples were systematically cleaned, unlocked, and air-dried. The color variation in dyed material and staining degree of the undyed fabric were evaluated using greyscale.

## 3. Results and discussion

**Figure 1:**
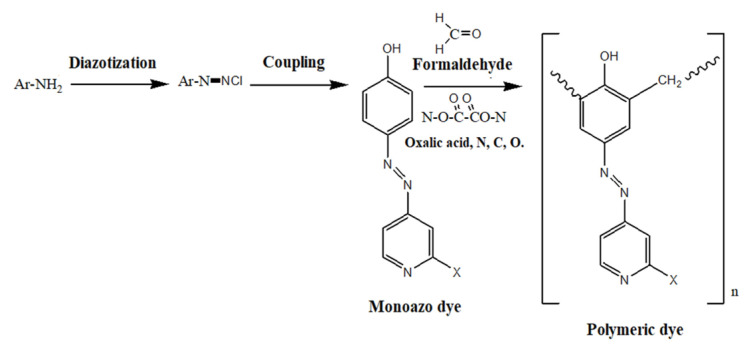
Scheme for synthetic routes for the polymeric dyes.

**Figure 2:**
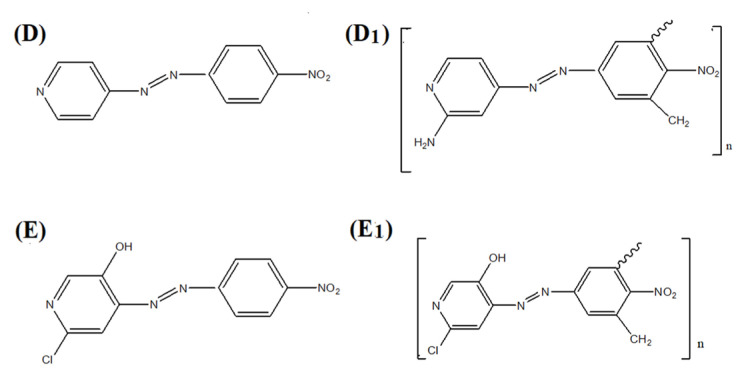
Monomeric dyes D: (4- Nitrophenylazo-3-aminopyridine), E: (4- Nitrophenylazo-3-chloro-6-hydroxypyridine), and Polymeric Dyes D_1_: Poly (4- Nitrophenylazo-3-aminopyridine), E_1:_ Poly (4- Nitrophenylazo-3-chloro-6-hydroxypyridine).

The synthetic routes for the polymeric dyes are depicted in the schemes presented as Figures 1 and 2.

### 3.1 Color, yield and melting points results of synthesized monomeric and polymeric dyes

The color, yield, and melting point of the synthesized monomeric/polymeric dyes are presented in Table 1. The dyes were yellow in color, with the polymeric dyes having deeper shades, and both the monomeric and polymeric dyes had good yield and low melting points. However, when comparing the monomeric dyes to the corresponding polymeric dyes, an increase in melting points and a deepening in color were observed, which could be due to an increase in molecular weight [29].

### 3.2 Solubility results of synthesized monomeric and polymeric dyes in various solvents

The solubility of monomeric and polymeric dyes in various solvents is shown in Table 2. The monomeric/polymeric dyes are soluble in acetone, ethanol, and methanol; however, they are insoluble in n-hexane, benzene, and water, which might be due to the polar character of some of the dyes and solvents. Their insolubility in an aqueous medium confirmed the dispersed nature of these dyes [29]. The solubility of monomeric/polymeric dyes in various solvents is presented in Table 2.

### 3.3 Viscosity results of synthesized monomeric and polymeric dyes in acetone

The dilute solution viscosity of the dyes was determined using Ostwald viscometer and presented in Table 3. As can be seen even at infinite dilution, polymeric dyes were found to have higher viscosity than their monomeric counterparts, indicating that the polymer molecule’s conjugation has increased. This clearly shows that polymeric dyes will be absorbed and remain fixed into the fiber matrix, and it is one of the reasons for polymeric dyes’ higher substantivity and fixation on fiber in comparison to monomeric dyes [6]. According to the Mark-Houwink-Sakurada equation, a substance’s viscosity is proportional to its molecular weight. The polymer is predicted to have a larger molecular weight than the monomer due to the increased repetition unit in the polymer chain, which leads to a higher molecular weight.


(1) $$[\eta ]=K{M_{\text{v}}}^{\alpha },$$

where η = viscosity, Mv = viscosity average molecular mass of the solution, K, α = constant for polymer/solvent at a given temperature [15, 16].

### 3.4 UV-visible analysis

**Figure 3:**
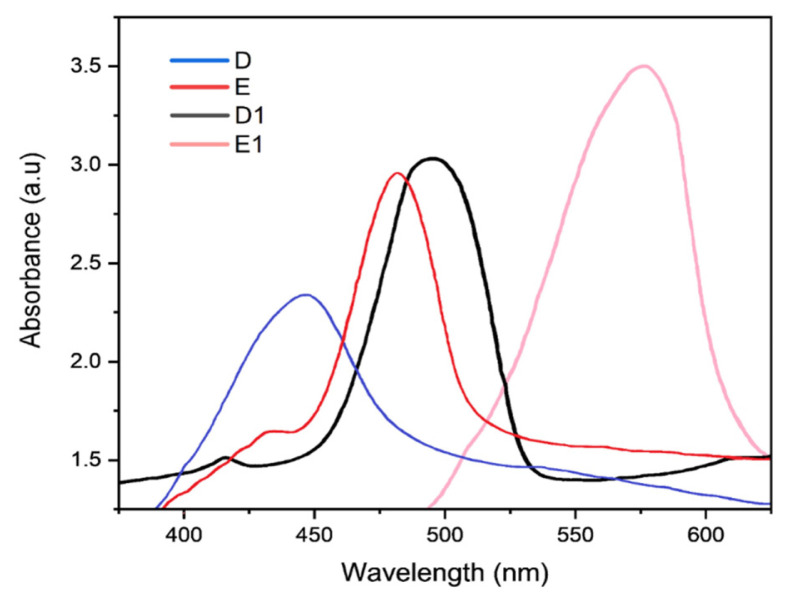
UV-visible spectra of heterocyclic monomeric dyes with corresponding polymeric dyes monomeric dye D, and E, corresponding polymeric dye D_1_ and E_1._

The wavelength of maximum absorption of the synthesized dyes can be seen in Table 4. The wavelength of maximal absorption (*λ*_max_) was determined using UV-visible analysis on both monomeric and polymeric dyes. The UV-vis spectra of the dyes displayed typical electronic spectra, with several absorptions in the UV area between 274 and 400 nm (referred as the B-band) and others in the visible range between 450 and 800 nm (referred as Q-band). The n-π transition of the dye molecules conjugated chain from the highest occupied molecular orbital (HOMO) to the lowest unoccupied molecular orbital (LUMO) produces the Q-band [11]. In general, all the dyes absorbed Q-band light, which is in the visible region of the spectrum, especially polymeric dyes, indicating an increase in molecular weight in comparison to monomeric counterparts. The intensity of the polymeric dyes color increases in comparison to their corresponding monomeric counterparts (monomeric dye **D** has yellow with (*λ*_max_) 440 nm while polymeric **D_1_** deep yellow color (*λ*_max_) 495 nm and monomeric dye **E** yellow (*λ*_max_) 487 nm while polymeric **E_1_** deep yellow color (*λ*_max_) 592 nm). Therefore, the shift of λ_max_ and deepened in color of the polymeric dyes compared to monomeric dyes as shown in Table 4, and depicted by Figure 3, indicates a bathochromic shift of the wavelength of maximum absorption. This shows an increase in conjugation and confirms that polycondensation has been achieved as reported previously [29].

The wavelength of maximum absorption of the synthesized dyes is given in Table 4.

### 3.5 FT-IR analysis

**Figure 4:**
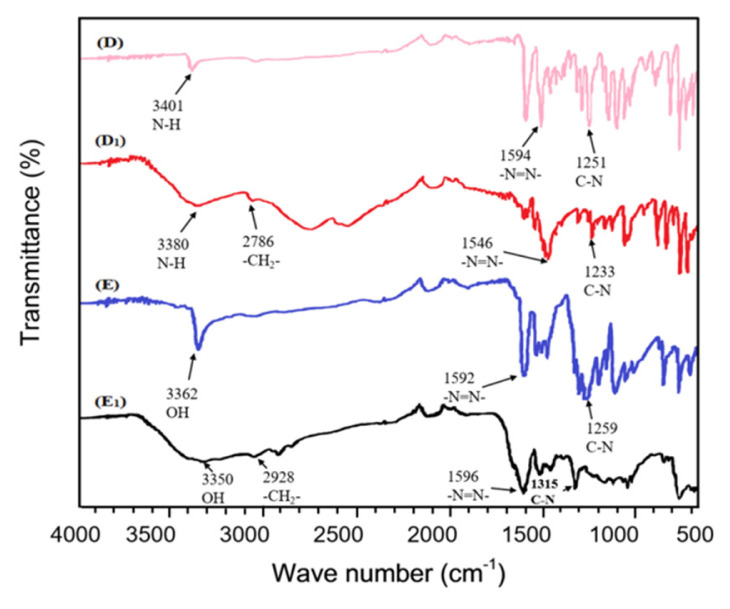
FTIR spectra of heterocyclic monomeric dyes with corresponding polymeric dyes monomeric dye D, and E, corresponding polymeric dye D_1_ and E_1_.

The FT-IR spectra of monomeric and polymeric dyes are presented in Figure 4. The absorption peaks of important functional groups are presented in Table 5. As can be seen in Figure 4 and Table 5, all the absorption peaks present in the synthesized polymeric dyes were found to be similar to those of the monomeric counterparts except that some shifts were observed for the prominent absorption peaks. The FT-IR spectra show the shift in the absorption peak of hydroxyl stretching vibration for the dye sample at 3362 cm^−1^ for monomeric dyes **E** to 3350 cm^−1^ for the polymeric dyes **E_1_**. The absorption peak at a region of 1546–1596 cm^−1^ was attributed to the presence of –N=N- group, and the spectral bands observed in the region of 1233–1315 cm^−1^ were allocated to (C-N). The only difference between the monomeric spectra and their corresponding high molecular weight counterparts is the presence of (**-CH_2_-)** asymmetric methylene absorption peak at 2786 and 2928 cm^−1^ for the heterocyclic polymeric dyes D_1_ and E_1_, respectively [6].

### 3.6 Fastness rating of the synthesized dyes on nylon and polyester

Comparative ratings of color fastness of the monomeric, polymeric dyes and commercial disperse dyes (terasil brilliant violet and terasil scarlet, brown) on nylon and polyester fibers were evaluated in accordance to the American Society for Testing and Materials (ASTM) and presented in Tables 6 and 7.

#### 3.6.1 Fastness to sublimation (hot pressing)

The dye’s resistance to sublimation treatment was tested using color staining on undyed white fiber and the results are presented in Tables 6 and 7. The results showed that the prepared and commercial dyes give good to excellent ratings (ranging from grade 3–4 to 5). Furthermore, the polymeric dyed polyester and nylon textiles had an exceptional hot-pressing (sublimation) rating. This may be ascribed to the development of long molecular weight compound and the existence of polar groups (OH, NH_2_) which causes the formation of intermolecular hydrogen bonding [27].

#### 3.6.2 Fastness to rubbing

The purpose of the test was to measure the degree of color transfer from the dyed fabrics surface to an adjacent undyed cloth surface by rubbing. Tables 6 and 7 show that all the dyes had good to excellent rubbing fastness values of grades 4 and 5, implying very good results using the greyscale method, but polymeric dyes were found to have an excellent fastness to rubbing in comparison to monomeric and commercial disperse dyes. This performance may be due to adequate dye absorption and fixation on the fibers using both high temperature and carrier dyeing methods [30].

#### 3.6.3 Fastness to washing

The wash fastness of the dyed nylon and polyester fabric treated with monomeric, polymeric, and commercial disperse dyes are presented in Tables 6 and 7, and varied from good to excellent. However, the fibers treated with polymeric dyes were found to have an excellent fastness to washing. This might be attributed to their fixing ability on the fabrics hence the dye molecules are absorbed onto the matrix of the fiber and remain fixed due to covalent and hydrogen bonding that exists between the dye molecules and the fiber [30].

#### 3.6.4 Fastness to light

The as-synthesized and commercial disperse dyes demonstrated good to excellent fastness to light, ranging from grade 6 to 8 according to the standard blue wool for color changes as presented in Tables 6 and 7. However, the polymeric dyes were found to have an excellent fastness to light. The presence of electron-withdrawing groups in the proximity of the azo group and the formation of high molecular weight leading to the intermolecular hydrogen bonding may be responsible for the light fastness [31]. The heterocyclic polymeric dyes undergo tautomerism which causes an increase in the light fastness because of the decrease in electron density associated with the azo group via hydrogen bonding which can lower the sensitivity towards photochemical oxidation [7].

Generally, the result reveals that polymeric dyes have a higher fastness or resistance external agencies compared to monomeric dyes and commercial disperse dyes, this improvement of fastness properties is a result of the formation of long polymer conjugation which makes the polymeric dyes be fixed on the fiber-matrix [30]. Moreover, the carrier dyeing technique was found to be the best method for dyeing nylon and polyester with polymeric dyes [32].

#### 3.6.5 Fastness ratings of the synthesized dyes on nylon and polyester dyed using carrier dyeing technique

The comparatives of fastness properties of monomeric, polymeric dyes and commercial disperse dyes (terasil brilliant violet and terasil scarlet brown) were assessed using the standard methods and presented in Table 6.

The hot pressing, rubbing, and washing fastness results for the monomeric, commercial disperse dyes and polymeric dyes on nylon and polyester vary from good to excellent, while their light fastness ranges from moderate to outstanding fastness [15] (Table 7). It has been observed from Table 7 that the heterocyclic azo disperse polymeric dyes (D_1_ and E_1_) have excellent hot pressing, rubbing, washing and light fastness properties when compared with their corresponding monomeric dyes (D, E) and commercial disperse dyes (C, and F), which also agreed with literature reported by Derkowska-Zielinska et al. [16].

#### 3.6.6 Fastness ratings of the synthesized dyes on nylon and polyester dyed using high temperature dyeing technique

The comparatives of color fastness properties of monomeric, polymeric dyes and commercial disperse dyes (terasil brilliant violet and terasil scarlet brown) on nylon and polyester fibers were determined via the standard methods and presented in Table 7.

As presented in Table 7, the hot-pressing fastness for monomeric, commercial disperse dyes and polymeric dyes ranges from moderate to very good, while the rubbing and washing fastness vary from good to excellent [15]. The light fastness of monomeric, commercial disperses dyes and polymeric dyes on nylon and polyester fabrics ranges from moderate outstanding fastness, the outstanding fastness occurs on heterocyclic polymeric dyes (D_1_ and E_1_) [16]. Generally, all polymeric dyes have better fastness properties compared to their corresponding monomeric dyes and commercial disperse dyes.

#### 3.6.7 Comparative discussion of present study and reported literature

The production of eco-friendly, high quality, cost-effective, high substantivity, fiber affinity with outstanding fastness properties polymeric dyes for coloration of textile and nontextile substrates is a very crucial topic of interest by many chemists recently. The application of polymeric dyes on the fiber and its substantivity is influenced by various parameters such as melting point, viscosity, yield, and fastness ratings on fibers. In this discussion, the main characteristics of as-synthesized polymeric dyes were compared to other relevant literature based on physical attributes and fiber fastness ratings. Viscosity is one of the crucial factors that confirms the effective development of polymeric dye; as can be seen, the current study findings demonstrate the production of a high viscous dye material with increased conjugation (molecular weight). This is supported by the formation of FTIR absorption peak at a region of 2786, 2928 cm^−1^ which is attributed to the methylene -CH_2_- (D_1_ and E_1_) linking two or more dye monomer molecules. They were found to have high yield and moderate melting point compared to the reported data in the literature. Even among the polymeric dyes, the once obtained from heterocyclic components was observed to have outstanding properties in comparison to other polymeric carbocyclic dyes. Moreover, the heterocyclic azo polymeric dyes produced in this study has unique properties, and when applied on nylon and polyester it was found to have an excellent fastness to light and wash in comparison to the once reported in the literature and commercial disperse dyes used in this research as control. This might be due to the fact that the good methods of their synthesis, application on fiber (carrier dyeing technique) improve the dye migration from the dye bath to the fiber matrix, fixation and affinity as presented in Table 8.

## 4. Conclusion

Polymerization of various disperse heterocyclic monoazo dyes synthesized through diazotization, coupling, and polycondensation reactions has been achieved. The resulting monoazo disperse monomeric and polymeric dyes were characterized as described, the monomeric, polymeric, and commercially available disperse dyes (terasil brilliant violet, terasil scarlet, brown) were applied on nylon and polyester fabrics using carrier and high-temperature dyeing techniques and fastness properties were assessed using blue wool and greyscale standards. The polymeric dyes were found to have better washing and light fastness (grade 8) compared to the monomeric (grades 4 and 6) and commercially (grades 4 and 6) available disperse dyes, this is due to the increase in conjugation which leads to the formation of high molecular weight compounds and intermolecular hydrogen bonding interactions between polar groups present in the polymeric dye molecules. Within the fiber molecule, a higher number of polar groups in the dye molecule result in increased dyeing affinity and improved fastness ratings in comparison to the fibers dyed with monomeric and commercial disperse dyes. When they are absorbed into the fiber matrix they become fixed and highly sustentative to the fiber through covalent and hydrogen bonding that exists between nylon and polyester fabrics with the dyes. The result of fastness properties reveals that carrier dyeing is better than the high-temperature dyeing technique because of the resistance of the dye on the fiber to washing and light which indicates how the dyes molecules are held in the fibers matrix. In the future study, it is recommended to synthesize heterocyclic azo polymeric dyes in situ (in the fiber matrix) and is expected to have very outstanding substantivity, fixation, migration, leveling and fastness properties due to the possibility of the formation of the polymeric dyes inside fiber matrix, which produces a stronger hydrogen and covalent bonding between the fiber and dyes molecules.

## Figures and Tables

**Table 1:** Color, percentage yield, and melting point of monomeric and polymeric dyes.

Dyes sample		Color	Yield (%)	Melting point (°C)
Monomeric	D	Yellow	87.2	112.6–121.2
	E	Yellow	73.3	136.0–137.0
Polymeric	D_1_	Deep yellow	76.6	134.0–144.5
	E_1_	Deep yellow	62.8	149.4–154.7

**Table 2:** Solubility of monomeric and polymeric dyes in various solvents.

Solvents	Monomeric dyes		Polymeric dyes	
	D	E	D_1_	E_1_
Water	SS	SS	SS	SS
Methanol	S	SS	SS	SS
Ethanol	S	SS	S	SS
Acetone	S	S	S	S
Ethyl acetate	SS	SS	SS	SS
Benzene	SS	SS	I	I
Toluene	SS	SS	SS	SS
n-hexane	I	I	I	I
Chloroform	SS	SS	SS	SS

S = Soluble; SS = Sparingly soluble; I = Insoluble.

**Table 3:** Viscosity of synthesized disperse monomeric and polymeric dyes in acetone using Ostwald viscometer.

Dye samples	Conc. (gdL^−1^)	Average flow time (s)	Relative viscosity (η_rel_)	Specific viscosity (η_sp_)	Intrinsic viscosity η_sp /_ C
Monomeric					
D	0.25	14.64	1.128	0.128	0.512
	0.50	14.92	1.149	0.149	0.298
	0.75	15.21	1.172	0.172	0.229
	1.00	15.47	1.192	0.192	0.192
E	0.25	14.52	1.119	0.119	0.476
	0.50	15.01	1.156	0.156	0.312
	0.75	15.31	1.180	0.180	0.240
	1.00	15.51	1.195	0.195	0.195
Polymeric					
D_1_	0.25	17.87	1.377	0.377	1.508
	0.50	18.19	1.401	0.401	0.802
	0.75	18.41	1.418	0.418	0.557
	1.00	18.61	1.434	0.434	0.434
E_1_	0.25	17.93	1.381	0.381	1.524
	0.50	18.42	1.419	0.419	0.838
	0.75	18.63	1.435	0.435	0.580
	1.00	18.91	1.457	0.457	0.457

**Table 4:** UV-visible results of various monomeric and polymeric dyes.

Dye samples		λ _max_ (nm)	Absorbance
Monomeric	D	440	2.434
	E	487	2.969
Polymeric	D_1_	495	3.128
	E_1_	592	3.471

**Table 5:** Fourier transformed infrared spectroscopy (FT-IR) result for both polymeric and monomeric dyes.

Functional groups (cm^−1^)	Monomeric dyes		Polymeric dyes	
	D	E	D_1_	E_1_
-N=N-	1594	1592	1546	1596
-OH	-	3362	-	3350
C-N	1251	1259	1233	1315
N-H	3401	-	3380	-
-CH_2_-	-	-	2786	2928

**Table 6:** Fastness ratings of the synthesized dyes on nylon and polyester fabrics using carrier dyeing method.

Dyes samples		Nylon				Polyester			
		Hot pressing	Rubbing	Washing	Light	Hot pressing	Rubbing	Washing	light
Monomeric	D	4–5	4–5	4–5	6	4–5	4–5	4–5	6
	E	4–5	4–5	4–5	6	4–5	4–5	4–5	6
Commercial disperse	C^a^	4–5	4	4–5	6	4	4–5	4	6
	F^b^	4	4	4	6	4–5	4–5	4	6
Polymeric	D_1_	5	5	8	8	5	5	8	8
	E_1_	5	5	8	8	5	5	8	8

a **C;** terasil brilliant violet,

b F; terasil scarlet brown.

**Table 7:** Fastness ratings of the synthesized dyes on nylon and polyester fabrics using high temperature dyeing method.

Dyes samples		Nylon				Polyester			
		Hot pressing	Rubbing	Washing	Light	Hot pressing	Rubbing	Washing	light
Monomeric	D	4	4–5	4	6	4–5	4–5	4	6
	E	4	4–5	4	6	3–4	4–5	4–5	6
Commercial disperse	C^a^	4–5	4–5	4–5	5	3–4	4–5	4	6
	F^b^	3–4	4	4–5	4	2–3	4–5	4	4
Polymeric	D_1_	5	5	7	8	5	5	7	7
	E_1_	5	5	7	7	5	5	7	7

a **C;** terasil brilliant violet,

b F; terasil scarlet brown.

Monomeric dye D and E corresponding polymeric dye D_1_ and E_1_; commercial disperse dyes **C =** terasil brilliant violet; F = terasil scarlet, brown.

**Table 8:** Comparison of basic properties of heterocyclic polymeric dyes and previous reported study.

Type of dyes	Fiber	Method of application	Fastness rating		Viscosity	Yield %	Melting point (ºC)	Ref
			Washing	Light				
Carbocyclic azo polymeric dyes	Polyester and nylon	High temperature and carrier dyeing	8 7	7 6–7	High	59.3	136.0–154.7	[11]
Water-soluble crosslinking polymeric dyes	Silk and cotton	Room temperature dyeing	4–5 4	3–4 3–4	-	-	-	[33]
1,4-Diamino anthraquinone polyamides	Polyester	High temperature dyeing	6 – 7	5	Low	54.5	265–267	[34]
Polymeric monoazo disperse dyes	Polyester	High temperature dyeing	4–5	5	Low	38.9	138–142	[6]
Polymeric heterocyclic azo pigment	-	High temperature dyeing	-	4	-	81	210–212	[7]
Polymeric colorants	Polyester	Carrier/high temperature dyeing	4	3	-	89	289	[15]
Crosslinking polymeric dyes	Silk Cotton	Crosslinking dyeing of polymeric dye	4 4	- -	- -	92	-	[35]
Polyvinylamine dye	Cotton Silk	High temperature dyeing	4 4–5	3–4 3–4	- -	56	-	[12]
Novel 1,2-diol containing azo polymeric dyes	Polyester and nylon	High temperature dyeing	-	4–5 4	- -	40	89	[13]
Heterocyclic azo polymeric dyes	Polyester and nylon	High temperature and Carrier dyeing	8 8	8 8	High	76.6	149.4–154.7	**Present study**
